# Nickel chloride administration prevents the growth of oral squamous cell carcinoma

**DOI:** 10.18632/oncotarget.25313

**Published:** 2018-05-08

**Authors:** Hirotaka Ota, Takashi Shionome, Hisashi Suguro, Satsuki Saito, Kosuke Ueki, Yoshinori Arai, Masatake Asano

**Affiliations:** ^1^ Department of Pathology, Nihon University School of Dentistry, Tokyo, Japan; ^2^ Division of Immunology and Pathobiology, Nihon University School of Dentistry, Tokyo, Japan; ^3^ Department of Partial Denture Prosthodontics, Nihon University School of Dentistry, Tokyo, Japan; ^4^ Department of Endodontics, Nihon University School of Dentistry, Tokyo, Japan; ^5^ Division of Advanced Dental Treatment, Dental Research Center, Nihon University School of Dentistry, Tokyo, Japan; ^6^ Division of Applied Oral Sciences, Nihon University Graduate School of Dentistry, Tokyo, Japan; ^7^ Nihon University School of Dentistry, Tokyo, Japan

**Keywords:** nickel chloride, oral squamous cell carcinoma, matrix metalloproteinase, nude mouse, nuclear factor-kappa B

## Abstract

The effect of NiCl_2_ on oral squamous cell carcinoma-derived cell line HSC3 was examined. Incubation with 1 mM NiCl_2_ significantly reduced the expression of MMPs at mRNA and protein levels. The *in vivo* orthotopic implantation model was established by injecting highly metastatic subcell line HSC3-M3 to nude mouse tongue. After 1 week of injection, mice were fed with or without 1 mM NiCl_2_-containing water for two to three weeks. Immunohistochamical examination revealed that MMP9 expression was drastically reduced in NiCl2-fed mice. By CT images, cancer mass was observed as a translucent area in control mice. In NiCl_2_-fed mice, much highly translucent area was observed within the translucent area. Histologically, this area corresponded to the necrotic area in the tumor mass. Real-time PCR analysis revealed the reduced expression of angiogenic factors such as IL-8 and VEGF mRNA in NiCl_2_-fed mice. To further examine the effect of NiCl_2_ on metastasis, human β-globin gene expression in regional lymphnodes was compared. The β-globin gene was totaly absent in NiCl_2_-fed mice. Moreover, various cancer metastasis-related genes were inhibited in NiCl_2_-fed mice by PCR array analysis. The results indicated that NiCl_2_ might be a promising new anti-cancer therapeutics for the oral cancer treatment.

## INTRODUCTION

Oral squamous cell carcinoma (OSCC) is the most frequently occurring malignant tumor in the oral cavity [1]. Aberrant activities of transcription factors in OSCC result in the augmented expression of several other factors that contribute to tumor progression [1]. NF-κB, one of the most studied and important transcription factors belongs to a family of five members: p50 (NFKB1), p52 (NFKB2), p65 (RELA), c-Rel (REL), and RelB (RELB) [2]. Upon activation, these factors translocate to the nucleus where they participate in the expression of the genes involved in inflammatory and immune responses, as well as in cell proliferation and survival [3]. NF-κB protein levels increase gradually from premalignant lesions to invasive cancer, indicating its role during the early stages of carcinogenesis [4–7]. In a previous study, we reported the inhibitory effect of Ni^2+^ ions on NF-κB activity wherein, the ions directly bind to NF-κB p50 subunit and inhibit its nuclear transport, thereby, inhibiting IL-8 secretion [8].

Matrix metalloproteinases (MMPs) are the most important players in extracellular matrix remodeling and more than 20 members of the MMP family have been characterized to date [9, 10]. MMPs belong to the zinc-dependent family of endopeptidases which are implicated in a variety of physiological processes [9, 10]. One of the most relevant functions of the MMPs is the degradation of physical barriers, such as basement membrane [11–13]. As the invasive potential of various cancers depends on the ability of tumor cells to degrade the basement membrane [14], MMPs have been targeted by several therapeutic agents [11]. MMP2 and MMP9, in particular, have the ability to cleave type IV collagen, the main component of the basement membrane, and contribute to the metastasis of tumor cells [11–13].

The expression of MMPs in OSCC has been studied extensively [5, 15]. MMP expression is partially dependent on NF-κB [16]; hence, we speculated that Ni^2+^ ions might be able to inhibit the expression of these proteinases in OSCC. The aim of the present study was to examine whether Ni^2+^ ions can inhibit MMP expression and thereby inhibit growth and metastasis in OSCC. Our findings suggest that Ni^2+^ ions may be considered as a novel candidate for cancer treatment.

## RESULTS

### Ni^2+^ inhibits MMP expression

**Figure 1:**
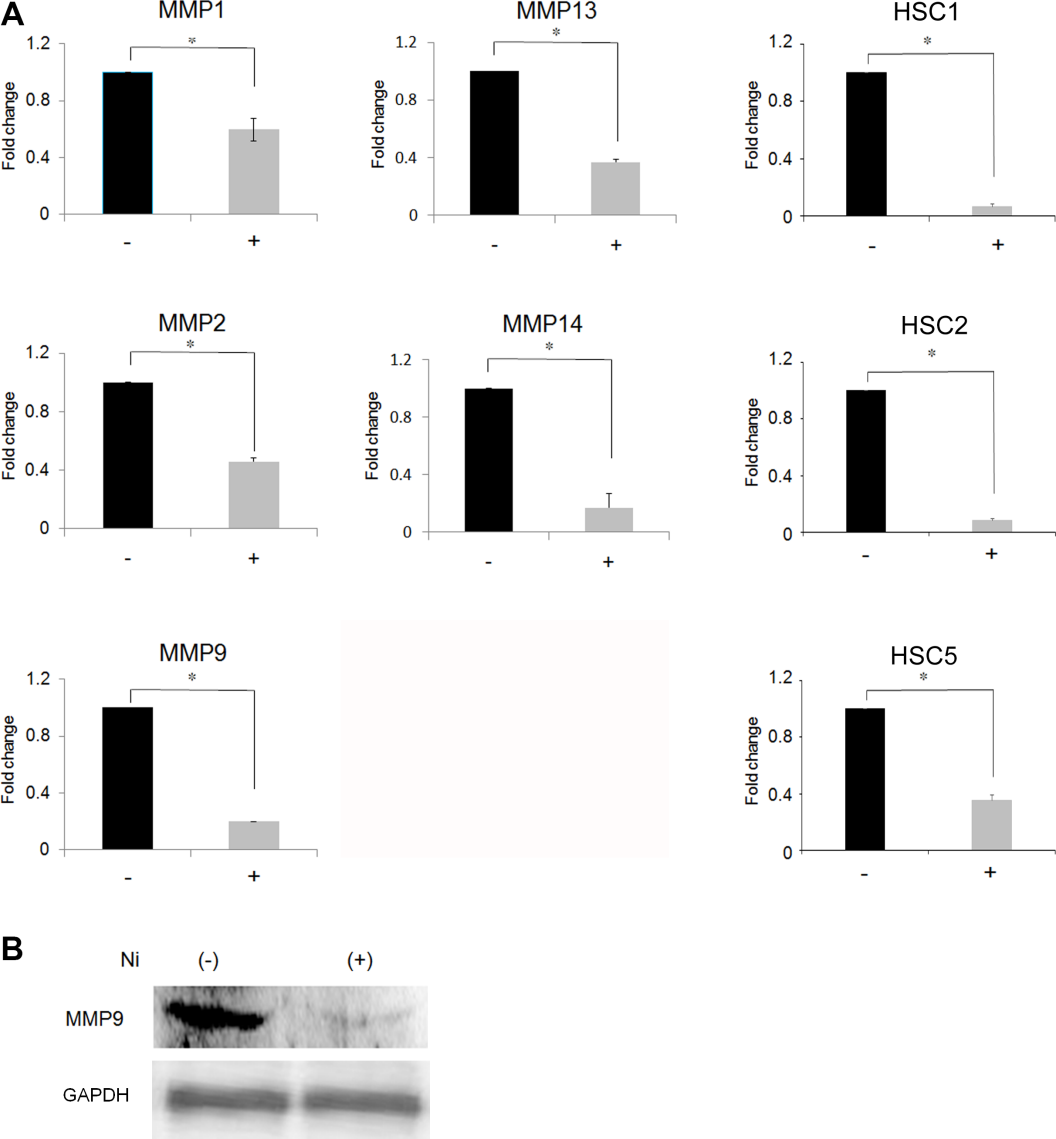
Inhibition of MMP expression following Ni^2+^-treatment (**A**) HSC3 cells and other squamous cell carcinoma cell lines (HSC1, 2 and 5) were cultured with or without NiCl_2_ (1 mM) for 24 h. The total RNA was purified and subjected to real-time PCR. MMPs expression in the cells cultured without Ni^2+^ was set as 1. The mean ± standard deviation (SD) of three separate experiments are shown. (^*^p < 0.05). (**B**) HSC3 cells were cultured with or without 1mM Ni^2+^ for 24 h. At the end of culture, the cell lysates were harvested and subjected to immunoprecipitation using anti-MMP9Ab (upper panel) or anti-GAPDH Ab (lower panel), followed by protein G-sepharose. The samples were separated with 10% SDS-PAGE and further subjected to Western blotting. Mouse anti-human MMP9 Ab or mouse anti-human GAPDH Ab followed by HRP-labelled goat anti-mouse IgG (H+L) Ab were used. The data is representative of three independent experiments.

We first attempted to examine the influence of Ni^2+^ ions on the expression of MMPs. For this purpose, HSC3 was cultured in the presence or absence of 1 mM NiCl_2_ for 24 h and MMP expression was examined by real-time PCR. MMP1, 2, 9, 13 and 14 were expressed in the HSC3 cells (Figure 1A). The expression level of each MMP without Ni^2+^ ions was set as 1 and the relative expression levels were compared in the cells stimulated with Ni^2+^ ions (Figure 1A). Ni^2+^ ions reduced the expression of all the MMPs in OSCC. The inhibition was most prominent for MMP9 and MMP14, with nearly 80% reduction when compared to the non-stimulated cells. On the other hand, a 35%–50% reduction was observed in MMP1, 2 and 13 levels. To confirm the results obtained above, we performed the same experiments using other cancer cell lines such as human skin-derived squamous cell carcinoma HSC1 and HSC5 and human OSCC HSC2. As shown in Figure 1A, MMP9 expression was drastically reduced in all cell lines by Ni^2+^ ions. MMP9 is known to play an intrinsic role in the invasive growth of cancer cells; therefore, the following experiments were performed with this particular peptidase. In order to examine the mechanisms involved in Ni^2+^ ions-mediated MMP9 reduction at the protein level, IP followed by Western blotting was performed. HSC3 was cultured with or without Ni^2+^ ions and the cell lysates were prepared. As shown in Figure 1B, a clear MMP9 band was detected when the cells were cultured without Ni^2+^ ions. In contrast, Ni^2+^ ions-treatment significantly reduced the intensity of the MMP9 band (Figure 1B).

### Ni^2+^ ions reduce MMP9 expression through NF-κB inhibition

**Figure 2:**
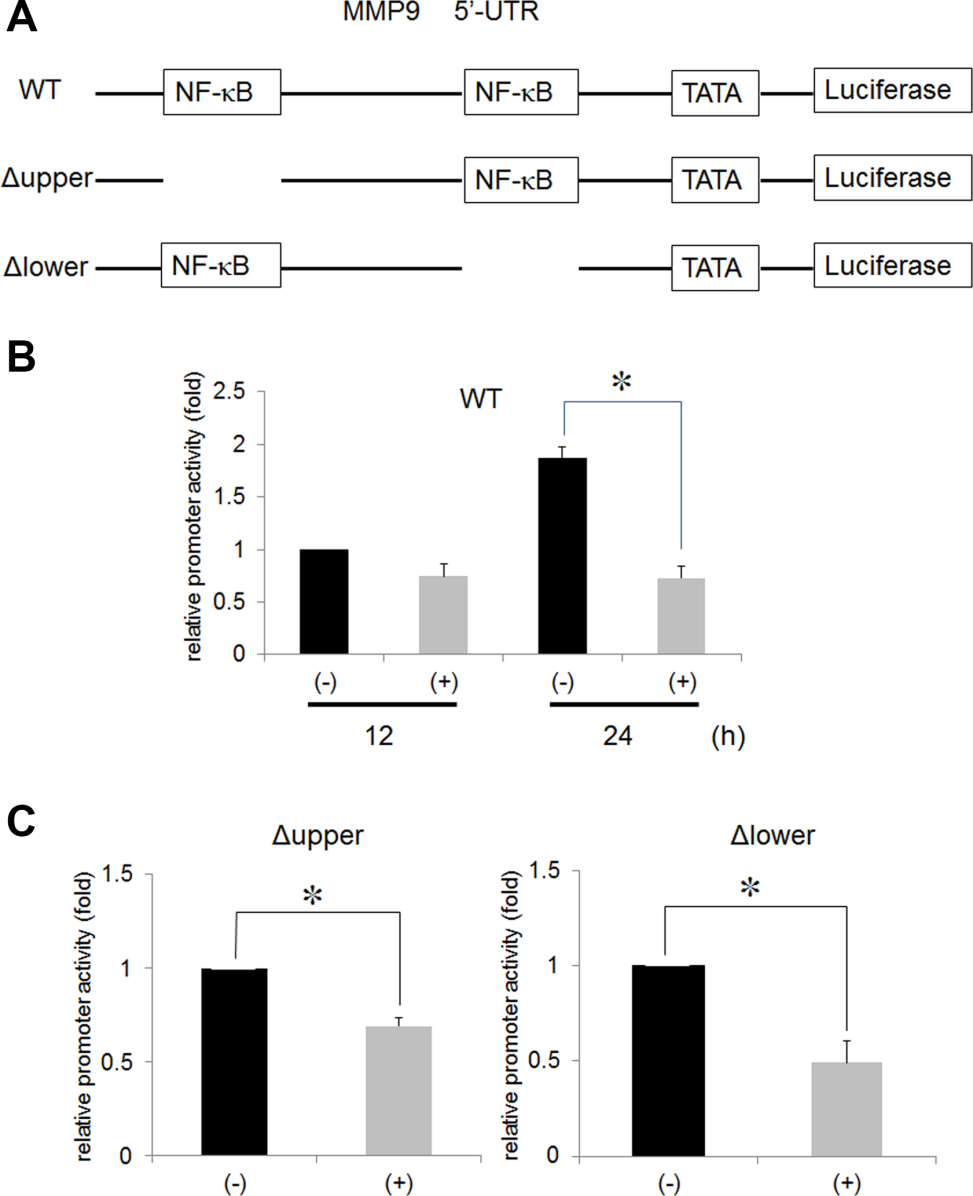
Ni^2+^ inhibited the NF-κB activity (**A**) Wild type 5′-UTR of the MMP9 gene was fused to luciferase reporter construct (WT). The upper (Δ upper) and lower (Δ lower) NF-κB binding sites were deleted by site-directed mutagenesis. (**B**) HSC3 cells were transfected with WT or (**C**) Δ upper or Δ lower along with pRL/CMV vector for 5 h. The cells were further cultured for 18 h and stimulated with or without 1 mM Ni^2+^ for 12 h or 24 h. At the end of stimulation, the cells were lysed with passive lysis buffer and luciferase activity was measured. The mean ± SD of three independent experiments was shown. (^*^p < 0.05).

In a previous study, Ni^2+^ ions were demonstrated to inhibit NF-κB activity [8]. To examine the contribution of NF-κB on Ni^2+^ ions-mediated MMP9 inhibition, we cloned the 5′-UTR of the MMP9 gene and performed a luciferase assay. The structure of the 5′-UTR is illustrated in Figure 2. We first transfected the wild type (pGL4-MMP9-wt) construct to HSC3 after which, the cells were stimulated with or without Ni^2+^ ions for 12 or 24 h. Luciferase activity of the cells without Ni^2+^ ions, after 12 h of culture, was set as 1 and compared with the Ni^2+^ ions-stimulated samples. Luciferase activity was slightly reduced at 12 h of stimulation (∼0.75); however, after 24 h stimulation, the activity was increased to 1.87 in the absence of Ni^2+^ ions and reduced to 0.73 in the presence of Ni^2+^ ions (Figure 2B). Based on the presumption that the reduced luciferase activity might be attributed to the inactivation of NF-κB by Ni^2+^ ions, we constructed NF-κB-deletion mutants lacking upper and lower NF-κB binding sites (Figure 2A). MMP9 5′-UTR encompasses two NF-κB binding sites. Each site was deleted and designated as Δ upper and Δ lower, respectively. In each case, luciferase activity in cells without Ni^2+^ ions stimulation was set as 1. The luciferase activity was reduced to 0.48 and 0.69 in the Δ upper and Δ lower mutants, respectively, in the cells stimulated with Ni^2+^ ions (Figure 2C). The results indicate that both upper and lower NF-κB-binding sites contribute to Ni^2+^ ions-mediated MMP9 reduction with a slight predominance of the upper region.

### Ni^2+^ ions in drinking water inhibits MMP9 expression

**Figure 3:**
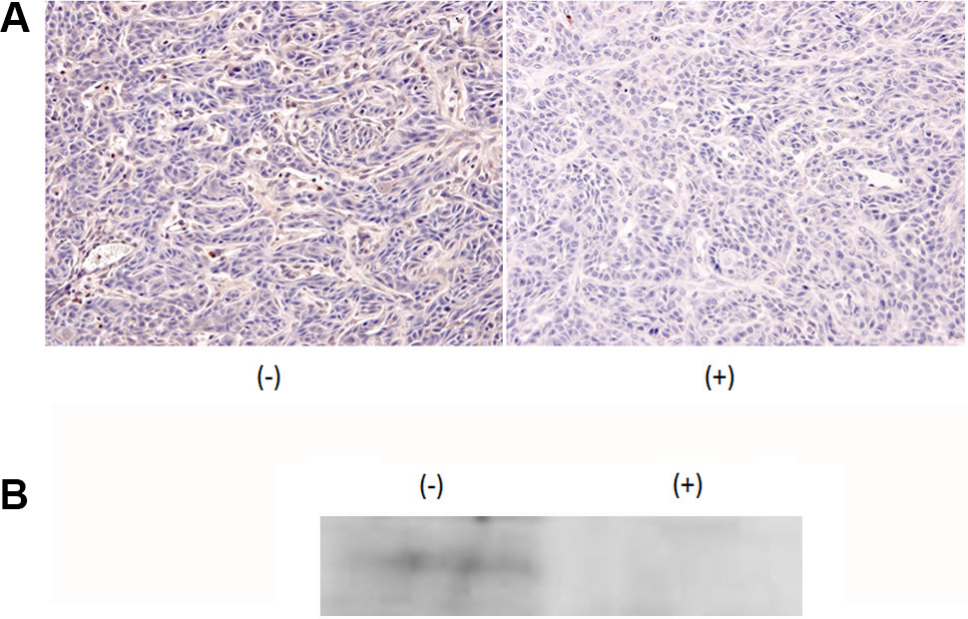
Addition of 1 mM NiCl_2_ in drinking water inhibited MMP9 expression HSC3 cells were xenografted to the tongue of nude mice. After tumor mass formation, mice were fed with regular drinking water (*n =* 5) or 1 mM NiCl_2_-containing water (*n =* 7) for 1 week. (**A**) The tissue was excised, formalin-fixed and embedded in paraffin. The expression of MMP9 was examined by immunohistochemical staining using anti-MMP9 Ab as the first Ab. (**B**) The excised tissue were minced, lysed with cell lysis buffer and subjected to IP-Western. Representatives of three independent experiments were shown for both (A and B).

To examine whether MMP9 expression is inhibited *in vivo,* cancer mass formation was induced by injecting HSC3 cells into the tongues of nude mice. Subsequently, the mice were fed with 1 mM NiCl_2_-containing water or regular water. The tumor mass size was measured at this time point and not significant difference between the two groups (regular water-fed group: 6.64 ± 0.63 mm^3,^ NiCl_2_-fed group: 6.07 ± 1.02 mm^3^) were observed. The tumor tissue was excised and subjected to anti-MMP9 staining. As shown in Figure 3A (left column), tumor cells showed dense staining for MMP9 in mice that were fed normal water; in contrast, significantly reduced intensity was observed in tumor cells derived from the mice fed with NiCl_2_-containing water (Figure 3A, right column). The reduction of MMP9 expression was confirmed by immunoprecipitation followed by western blotting (Figure 3B). Significant MMP9 reduction was observed in mice fed with NiCl_2_-containing water. These results indicated that Ni^2+^ ions can reduce MMP9 expression *in vivo*.

### Cancer growth inhibition

**Figure 4:**
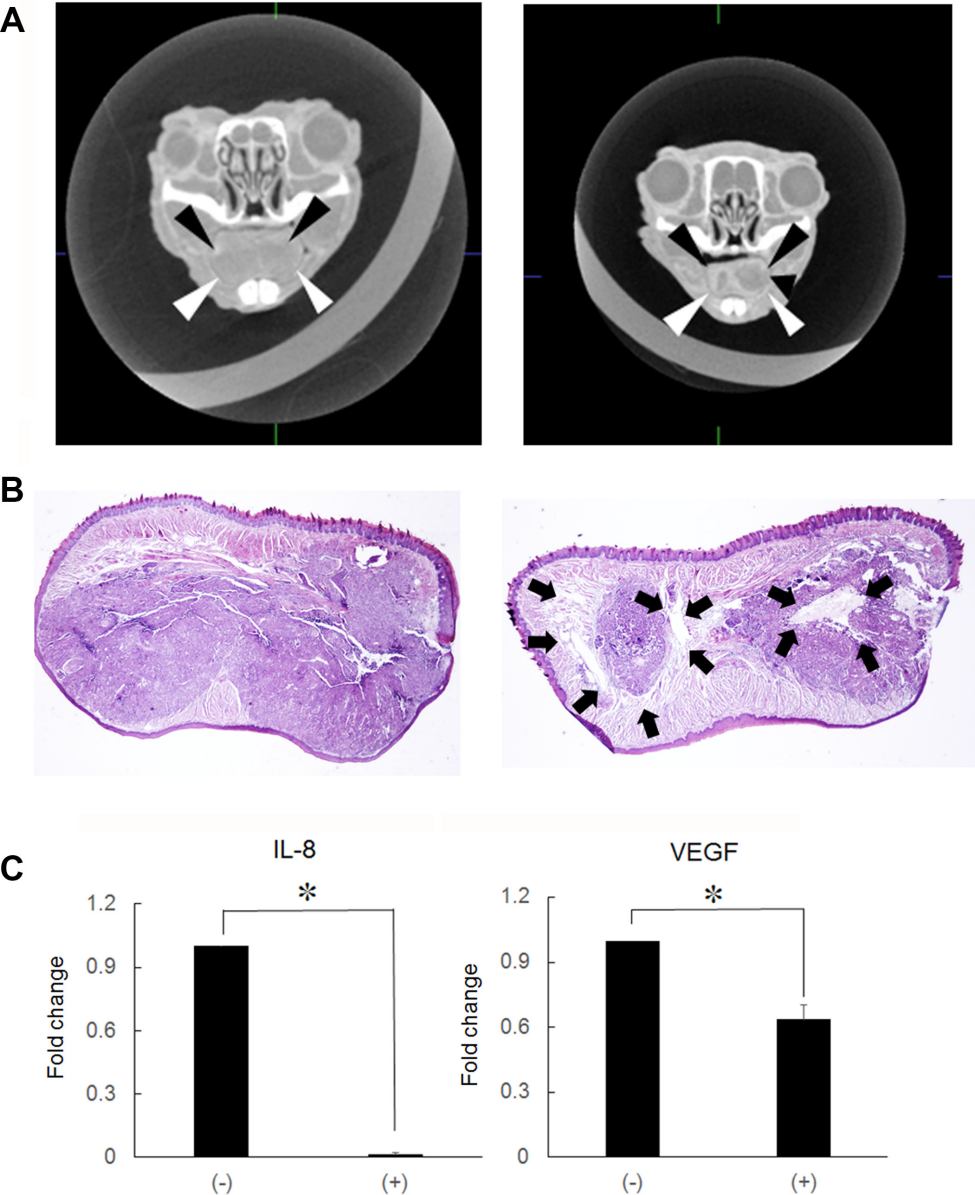
Production of hyper-lucent areas within the cancer mass (**A**) HSC3-M3 cells (5 × 10^5^/30 μl) were injected into nude mice tongue. After cancer mass formation (1 week), mice were fed with (*n =* 5) or without 1 mM NiCl_2_-containing water (*n =* 7) for 2 weeks. A contrast agent was injected into the tail vein and CT images of the primary regions were obtained. Left panel: regular water, arrowheads indicate the translucent area. Right panel: NiCl_2_, The hyper translucent area can be observed in the translucent area. The representative of 5 (non-fed) and 7 (Ni2+-fed) different images was shown. (**B**) The primary region was excised, fixed and embedded in paraffin. The specimens were stained by H&E staining. Left panel: regular water. Right panel: NiCl_2_-containing water. The arrows indicate the necrotic area. Representative data of at least five different experiments for each group are shown. (**C**) RNA was extracted from the tongue tissues and subjected to real-time PCR to examine IL-8 and VEGF expression. The mean ± SD of three separate experiments are shown. (^*^p < 0.05).

Growth and invasive potential of tumor cells is dependent on the spontaneous activity of transcription factors, such as NF-κB [1]. If Ni^2+^ ions inhibit NF-κB activity, Ni^2+^ ions might be applied as anti-cancer reagent. To examine this possibility, HSC3-M3 cells (5 × 10^5^), the highly metastatic subclone of HSC3, was injected in nude mice tongue. A contrast agent was injected and CT images of the primary regions were obtained. As shown in Figure 4A (frontal section), the cancer occupied the entire region of the tongue and presented as a radiolucent area with relatively clear borders in the regular water-fed group. The area was uniform in translucency and showed the so-called ball-in-hands image (Figure 4A, left panel). On the other hand, in the Ni^2+^ ions-fed group, a hyper-radiolucent area was observed within the radiolucent area (Figure 4A, right panel). The primary region was further examined histologically by HE staining. Solid cancer nests were noted in the regular water-fed group, (Figure 4B, left panel). In contrast, in the Ni^2+^ ions-fed group, necrotic areas were observed within the cancer nests (Figure 4B, right panel); this may be attributed to a reduction in angiogenic activity in the Ni^2+^ ions-fed group. Based on this assumption, we compared the expression of the angiogenic factors IL-8 and VEGF between the Ni^2+^ ions-fed and non-fed groups of mice. Tumor mass was excised and subjected to real-time PCR for IL-8 and VEGF expression. In the Ni^2+^ ions-fed group, significant reductions in both genes (∼100-fold and ∼1.5 -fold for IL-8 and VEGF, respectively) were observed (Figure 4C).

### Anti-metastatic effect of Ni^2+^ ions

**Figure 5:**
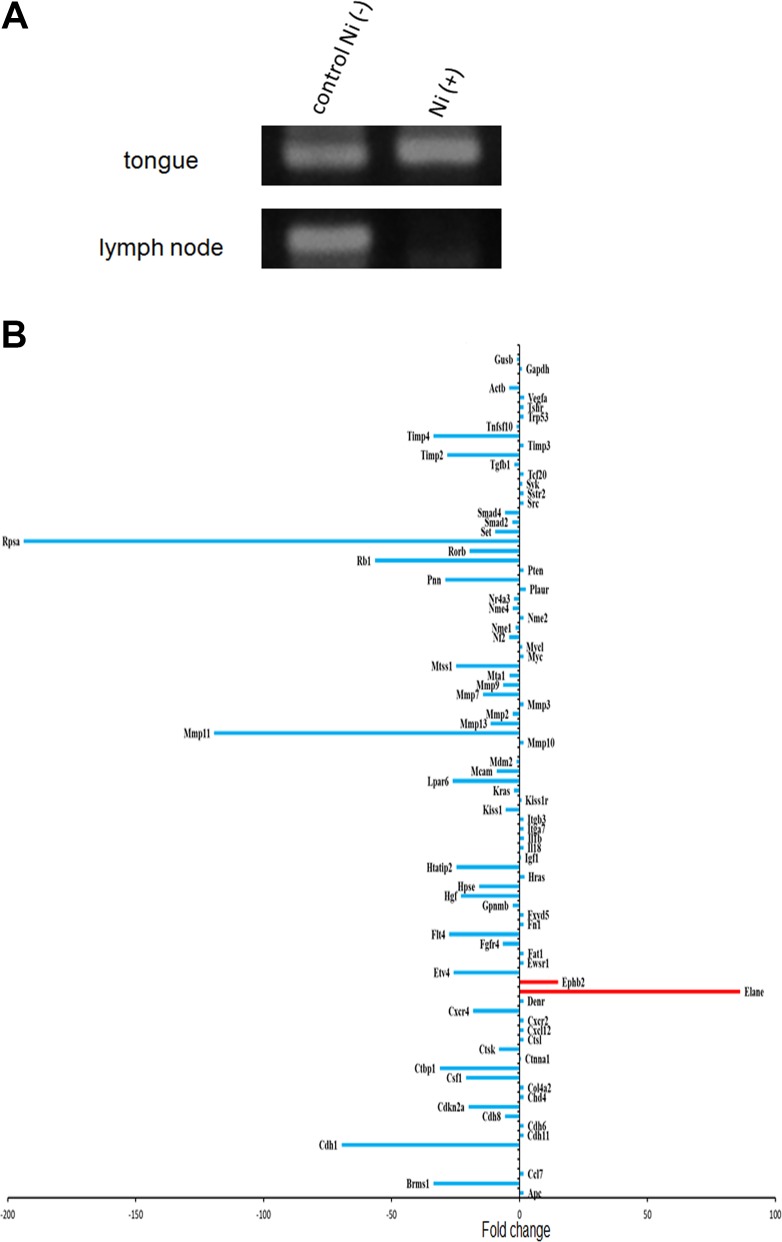
Anti-metastatic effect of Ni^2+^ (**A**) After 2 weeks of breeding with 1 mM NiCl_2_-containing water or regular water, primary tongue regions (upper panel) and lymph nodes (lower panel) were excised from 6 mice of each group. Genomic DNA was purified and subjected to PCR for β-globin gene. The representative of 6 separate experiments were shown. (**B**) RNA was purified from the tongue tissues obtained from nude mice fed with (*n =* 3) or without NiCl_2_-containing water (*n =* 3). PCR array was performed with RT^2^ Profiler PCR Array kit (QIAGEN) to examine the expression levels of the cancer metastatic genes. Expression levels of each gene relative to those in the Ni^2+^ non-fed control mice are shown. The representative of 2 different experiments were shown.

In order to examine the anti-metastatic effect of Ni^2+^ ions, regional lymph nodes were obtained from the mice and subjected to nested-PCR for β-globin gene detection. As HSC3-M3 cells are of human origin, detection of the human β-globin gene indicates the establishment of metastasis. Similar amounts of β-globin gene were detected in the primary tongue regions in both groups (Figure 5A, upper panel). On the other hand, a drastic reduction in β-globin gene expression was noted in the Ni^2+^ ions-fed group (Figure 5A, lower panel) when compared with the Ni^2+^ ions-unfed control group (Figure 5A).

### PCR array analysis

The influence of Ni^2+^ ions on the expression of cancer metastatic genes was further examined. RNA was purified from the cancer mass obtained from the Ni^2+^ ions-fed and -unfed mice, and subjected to PCR array analysis. The list of the genes analysed were shown in Table 1. Consistent with the data shown in Figure 1, MMP2, 3, 7, 9, 11 and 13 expressions were drastically reduced in the Ni^2+^ ions-fed group (Figure 5B). Except for the significant increase in elastase and Eph receptor B2 (epht2) expression (Figure 5B), most of the metastasis-related genes were reduced in the Ni^2+^ ions-fed group (Figure 5B).

**Table 1:** The genes analysed in this study

Official Symbol	Official Full Name	Official Symbol	Official Full Name
Apc	adenomatosis polyposis coli	Kras	Kirsten rat sarcoma viral oncogene homolog
Brms1	breast cancer metastasis-suppressor 1	Lpar6	lysophosphatidic acid receptor 6
Cd7	chemokine (C-C motif) ligand 7	Mcam	melanoma cell adhesion molecule
Cdh1	cadherin 1	Mdm2	transformed mouse 3T3 cell double minute 2
Cdh11	cadherin 11	Mmp10	matrix metallopeptidase 10
Cdh6	cadherin 6	Mmp11	matrix metallopeptidase 11
Cdh8	cadherin 8	Mmp13	matrix metallopeptidase 13
Cdkn2a	cyclin-dependent kinase inhibitor 2A	Mmp2	matrix metallopeptidase 2
Cdh4	chromodomain helicase DNA binding protein 4	Mmp3	matrix metallopeptidase 3
Col4a2	collagen, type IV, alpha 2	Mmp7	matrix metallopeptidase 7
Csf1	colony stimulating factor 1	Mmp9	matrix metallopeptidase 9
Ctbp1	C-terminal binding protein 1	Mta1	metastasis associated 1
Ctnna1	catenin (cadherin associated protein), alpha 1	Mtss1	metastasis suppressor 1
Ctsk	cathepsin K	Myc	myelocytomatosis oncogene
Ctsl	cathepsin L	MycI	v-myc avian myelocytomatosis viral oncogene lung carcinoma derived
Cxcl12	chemokine (C-X-C motif) ligand 12	Nf2	neurofibromin 2
Cxcr2	chemokine (C-X-C motif) receptor 2	Nme1	NME/NM23 nucleoside diphosphate kinase 1
Cxcr4	chemokine (C-X-C motif) receptor 4	Nme2	NME/NM23 nucleoside diphosphate kinase 2
Denr	density-regulated protein	Nme4	NME/NM23 nucleoside diphosphate kinase 4
Elane	elastase	Nr4a3	nuclear receptor subfamily 4, group A, member 3
Ephb2	Eph receptor B2	Plaur	plasminogen activator, urokinase receptor
Etv4	ets variant 4	Pnn	pinin
Ewsr1	Ewing sarcoma breakpoint region 1	Pten	phosphatase and tensin homolog
Fat1	FAT atypical cadherin 1	Rb1	RB transcriptional corepressor 1
Fgfr4	fibroblast growth factor receptor 4	Rorb	RAR-related orphan receptor beta
Flt4	FMS-like tyrosine kinase 4	Rpsa	ribosomal protein SA
Fn1	fibronectin 1	Set	SET nuclear oncogene
Fxyd5	FXYD domain-containing ion transport regulator 5	Smad2	SMAD family member 2
Gpnmb	glycoprotein (transmembrane) nmb	Smad4	SMAD family member 4
Hgf	hepatocyte growth factor	Src	Rous sarcoma oncogene
Hpse	heparanase	Sstr2	somatostatin receptor 2
Hras	Harvey rat sarcoma virus oncogene	Syk	spleen tyrosine kinase
Htatip2	HIV-1 Tat interactive protein 2	Tcf20	transcription factor 20
Igf1	insulin-like growth factor 1	Tgfb1	transforming growth factor, beta 1
II18	interleukin 18	Timp2	tissue inhibitor of metalloproteinase 2
II1b	interleukin 1 beta	Timp3	tissue inhibitor of metalloproteinase 3
Itga7	integrin alpha 7	Timp4	tissue inhibitor of metalloproteinase 4
Itgb3	integrin beta 3	Tnfsf10	tumor necrosis factor (ligand) superfamily, member 10
Kiss1	KiSS-1 metastasis-suppressor	Trp53	transformation related protein 53
Kiss1r	KISS1 receptor	Tshr	thyroid stimulating hormone receptor
		Vegfa	vascular endothelial growth factor A

## DISCUSSION

For effective tumor growth and invasion, the surrounding stroma must be degraded by the cancer cells [17]. One of the most important players involved in this process are the MMPs. MMP gene expression is partly regulated by NF-κB [18]. As Ni^2+^ ions were shown to inactivate NF-κB in our previous study [8], herein, we first examined the effect of Ni^2+^ ions on MMP expression. Expression of MMP1, 2, 9, 13 and 14 in OSCCs was confirmed by real-time PCR. The promoters of all these MMPs contain the NF-κB binding site; therefore, Ni^2+^ ions were expected to bind to these sites and inhibit the expression of these proteinases [18]. Consistent with our expectation, significant reductions in MMP expression were observed. Nevertheless, small amount of the mRNA expression was remained. MMP promoter conformation is classified into three different categories based on the existence of the TATA box and activator protein-1 (AP-1) sites [19]. AP-1 is a chief cis-acting element for the induction of MMPs, and the importance of both AP-1 and NF-κB on 12-O-tetradecanoylphorbol-13-acetate (TPA)-induced MMP9 expression has been reported previously [20]. Incomplete reduction of MMPs by Ni^2+^ ions might be attributed to the contribution of AP-1 and/or other transcription factors in OSCCs. In the cases of MMP2 and 14, however, the AP-1 sites are absent. Thus, Ni^2+^ ions -mediated MMP reduction mechanisms should be clarified in more detail.

Based on the above results, we presumed that the Ni^2+^ ions could exert inhibitory effects *in vivo.* A 2-week administration of NiCl_2_-containing water did not show any histological abnormalities in mouse organs (data not shown). This observation was consistent with a previously published report [21]. Although highly carcinogenic, Ni^2+^ ions are a weak mutagen with no direct contributions to the mutation process [22, 23]. MMP9 expression in implanted cancer cells has been examined by immunostaining. Surprisingly, MMP9 levels had decreased significantly in the 1 mM NiCl_2_-fed mice. In accordance with these results, significant reduction in the formation of metastatic lesions in regional lymph node were noted in the 1 mM NiCl_2_-fed mice in the present study. Ni^2+^ ions were found to bind directly to the NF-κB p50 subunit [8]; hence, in order for them to have an effect the ions must be incorporated into the cytoplasm. This can be done by three different methods: 1) diffusion across the cell membrane, 2) transport via calcium and ion channels, and 3) phagocytosis [22, 23]. Transport of Ni^2+^ ions has been inhibited by intestinal Caco-2 cells in the presence of Fe^2+^ [24]. Further investigations revealed the contribution of the proton-coupled divalent cation transporter (DCT1 and Nramp2) during this process [25, 26], which has a broad substrate range. Ni^2+^ ions are intrinsic nutrients for some bacteria, and must be taken up via a nickel transporter localized in the periplasm, which is composed of five different genes (Nik A to E), and allow for the active transport of the ions into the cytoplasm [27–29]. To gain insights into phagocytic Ni^2+^ ions-incorporation, HSC3 cells were pre-incubated with phagocytosis inhibitor, monodansylcadaverine and rottelerin. The effects of these inhibitors were assessed by measuring IL-8 concentrations in the culture supernatants; however, neither of them exerted any inhibitory effect (Supplementary Figure 1). An alternative mechanism should be receptor-mediated internalization. Ni^2+^ ions are chief drivers of contact allergy. Nonetheless, the specific interacting receptor has not been identified so far. Recently, human toll-like receptor 4 (TLR4) was demonstrated to interact with Ni^2+^ ions through histidine residues in the extracellular region [30]. The routes of Ni^2+^ ions-incorporation need to be examined in future.

In our previous study, Ni^2+^ ions were shown to bind directly to a histidine cluster in the Rel-homology domain (RHD) of the NF-κB p50 subunit [8]. RHD encompasses the nuclear localization sequence (NLS) in its most C-terminal region [31]. The NLS is recognized by its receptor importin α and this interaction is indispensable for the nuclear translocation of the NF-κB complex [32]. Moreover, importin α was originally identified by its Ni^2+^ ion binding ability [33]. It might be speculated that the binding of Ni^2+^ ions to p50 or importin α might modify its three-dimensional structure and prevent the recognition between importin α and p50 NLS.

Histological examinations revealed the formation of a necrotic area within the cancer mass in NiCl_2_-fed mice. The formation of this structure, which presented as a hyper-translucent area in CT images, might be due to the lack of angiogenic factors such as IL-8 and VEGF [34]. In HSC3 cells, IL-8 is constitutively produced under the control of NF-κB [8]. Ni^2+^ ions administration might reduce the production of angiogenic factors through inactivation of NF-κB. Consistent with this hypothesis, in the Ni^2+^ ions-fed group, decreased expression of IL-8 and VEGF mRNA was observed. Moreover, PCR array analysis, which focused on the genes related to cancer metastasis, confirmed the reduced expression of several genes. Only Elane and Ephb2 genes were upregulated by Ni^2+^ ions, the biological significance of which should be clarified in another study.

Metastatic activity was compared by detecting the β-globin gene. Although equivalent expressions were detected in the primary tongue region, β-globin gene was not detected in the regional lymph node in the Ni^2+^ ions-fed group. It has been reported that nickel compounds induce apoptosis in various cells [35–39]. For instance, in an attempt to elucidate the mechanisms underlying the anti-apoptotic effect of NiCl_2_, Yang et al. treated NiCl_2_-transformed bronchial epithelial cells with 1.5 mM NiCl_2_ to induce apoptosis [39]. They observed a substantial degree of apoptosis following NiCl_2_ treatment, which was dependent on the expression levels of anti-apoptotic gene such as Bcl-2 and Bcl-xL [39]. However, in this report, transformed cells were established by treating the cells with lower concentrations of NiCl_2_ [39]. These data are consistent with our observations in terms of the NiCl_2_ concentration used during the experiments (mM order of NiCl_2_ was used in both studies). The concentration of NiCl_2_ might be an important factor that influences the fate of the cell.

In conclusion, the present study illustrates the inhibitory effect of Ni^2+^ ions on growth in orthotopically implanted cancer cells. Further investigations are required to understand the fundamental mechanisms involved in this process. However, NiCl_2_ might be prove to a promising candidate for cancer therapeutics.

## MATERIALS AND METHODS

### Reagents

Nickel chloride (NiCl_2_) was purchased from Sigma (St. Louis, MO). Antibodies (Ab) against human MMP9 and glyceraldehyde-3-phosphate dehydrogenase (GAPDH) were purchased from Santa Cruz (Santa Cruz, CA). Horseradish peroxidase (HRP)-conjugated goat anti-rabbit and anti-mouse IgG (H+L) Abs were purchased from Jackson ImmunoResearch (West Grove, PA, USA).

### Cell culture and Ni^2+^ ions stimulation

The human OSCC cell lines (HSC3 and its subclone HSC3-M3 and HSC2) and human skin-derived squamous cell carcinoma cell lines (HSC1 and HSC5) [40] were obtained from Health Science Research Resources Bank (Osaka, Japan). HSC3, HSC3-M3 and HSC2 were maintained in RPMI1640. HSC1 and HSC5 were maintained in Dulbecco’s minimum essential medium (DMEM) and Iscove’s Modified Dulbecco’s Medium (IMDM), respectively. Each medium was supplemented with 10% FCS, 50 μg/ml streptomycin and 50 U/ml penicillin (10% FCS-RPMI, 10% FCS-DMEM and 10% FCS-IMDM). To test Ni^2+^ ion stimulation, 2 × 10^5^ cells were plated in a 24-well dish on the day before the experiment. The cells were washed twice with 10% FCS-RPMI and further cultured in the presence or absence of 1 mM Ni^2+^ ions.

### Real time-polymerase chain reaction (PCR) and PCR array analysis

Total RNA was purified using RNeasy mini kit (QIAGEN, Tokyo, Japan). cDNA was synthesized with Superscript III reverse transcriptase (Invitrogen, San Diego, CA) and subjected to real-time PCR [41]. Real-time PCR was performed using LightCycler nano (Roche, Tokyo, Japan) with SYBR green (TaKaRa, Tokyo, Japan). The primers used in this study are listed in Table 2. RT^2^ Profiler PCR Array kit (QIAGEN) was used for PCR array analysis.

**Table 2:** The primers used in this study

Primer		Sequence	
MMP9	Forward	5′-GGG ACG CAG ACA TCG TCA TC-3′	Real-time PCR
	Reverse	5′-TCG TCA TCG TCG AAA TGG GC-3′	
β-actin	Forward	5′-GGA GCA AGT ATC TTG ATC TTC-3′	
	Reverse	5′-CCT TCC TGC GCA TGG AGT CCT G-3′	
IL-8	Forward	5′-CCA GCC ATC AGC CAT GAG GGT-3′	
	Reverse	5′-GGA GCC CTT TCT GAA TCC GCA-3′	
VEGF	Forward	5′-GCA CCC ATG GCA GAA GG-3′	
	Reverse	5′-CTC GAT TGG ATG GCA GTA GCT-3′	
MMP9	Forward	5′-GTG GAA TTC CCC AGA CTT GCC TA-3′	Luciferase assay
	Reverse	5′-GGT GAG GGC AGA GGT GTC TGA-3′	
GH20	Forward	5′-GAA GAG CCA AGG ACA GGT AC-3′	nested-PCR
GH21	Reverse	5′ GGA AAA TAG ACC AAT AGG CGA-3′	
KM29	Forward	5′-GGT TGG CCA ATC TAC TCC CAG G-3′	
KM38	Reverse	5′-TGG TCT CCT TAA ACC TGT CTT G-3′	

### Immunoprecipitation (IP) and Western blotting

HSC3 cells were stimulated with or without 1 mM Ni^2+^ ions for 24 h. After stimulation, the cells were washed twice with ice cold PBS and lysed with 500 μl of cell lysis buffer (50 mM Tris-HCl, pH 7.5, 150 mM NaCl and 0.5% TritonX-100). After centrifugation, the samples were transferred to new tubes and rotated with 1 μl of rabbit anti-human MMP9 Ab for 24 h at 4°C. Protein G-sepharose (10 μl, GE Healthcare, USA) was added to the samples, which were then rotated for another 2 h. At the end of incubation, the samples were washed with ice cold PBS by centrifugation, loaded on to 10% SDS-PAGE and subjected to Western blotting as described previously [41]. The primary Ab against MMP9 was diluted to ×1,000 with 1% BSA-PBST (0.1% tween-20/PBS). The secondary HRP-goat anti-mouse IgG (H+L) was diluted to ×10,000 with 1% BSA-PBST. The bands were detected using an ECL kit (GE Healthcare).

### Cloning of 5′-untranslated region (UTR) of MMP9 gene and plasmid construction

The luciferase construct containing a 626 bp fragment of the MMP9 gene promoter spanning from –626 to –1 (+1 corresponds to the A of the ATG translation initiation codon) was amplified using the primers listed in Table 1. The amplified fragment was subcloned into the Hind III and Kpn I sites of the pGL4-basic vector (Promega, Tokyo, Japan). This reporter construct was designated as the wild type (pGL4-MMP9-wt). Using this plasmid as a template, deletion mutants lacking upper and lower NF-κB binding sites were constructed uing the QuickChange Site-directed Mutagenesis kit (Agilent Technologies, Tokyo, Japan), and designated as Δ upper and Δ lower, respectively.

### Luciferase assay

HSC3 cells were plated in 48-well culture plates at a density of 1 × 10^5^ cells/well. The cells were washed twice with OPTI-MEM and transfected with 1 μg of reporter plasmids (pGL4-MMP9-wt, Δ upper or Δ lower) using the Lipofectamin transfection method (Invitrogen, Tokyo, Japan). After 5 h of transfection, the cells were washed with 10% FCS-RPMI and further cultured for 18 h. Then, the cells were washed and either left unstimulated or stimulated with 1 mM Ni^2+^ ions for 12 and 24 h. After stimulation, the cells were lysed with 1× passive lysis buffer (Promega, Tokyo, Japan) and cell lysates were collected. Transfection efficiency was normalized to renilla luciferase activity by co-transfection with the pRL/CMV vector (Promega). Both firefly and renilla luciferase activities were determined using the Dual-Luciferase Reporter Assay System (Promega). Luminescence was measured on a Lumat LB9507 luminometer (Berthold, Bad Wildbad, Germany).

### Orthotopic implantation and immunohistochemistry

HSC3-M3 cells (5 × 10^5^/50 μl PBS) were injected into the right edge of the tongue of male nude mice. (CLEA, Tokyo, Japan). Experimental protocols were approved by the Nihon University School of Dentistry Animal Ethical Committee and performed according to legal requirements (AP14D030). Tumor formation was monitored every week. After cancer mass formation, the mice were fed with regular drinking water or 1 mM NiCl_2_-supplemented drinking water for 7 or 14 days. The mice were sacrificed by CO_2_ inhalation and fixed by perfusion with formalin. The cancer tissue was excised and embedded in paraffin. Four-micrometer-thick sections were prepared, deparaffinised in xylene and rehydrated with 100% ethanol. Endogenous peroxidase activity was inactivated with 0.3% hydrogen peroxide in methanol for 20 min at room temperature (RT). The sections were boiled in 10 mM citrate buffer (pH 6.0) for 20 min and cooled down. To block non-specific binding, the sections were incubated with 1% BSA-PBS at RT for 1 h. The blocking solution was removed, and the first Ab was applied following which, the samples were incubated at RT for 1 h. Negative control studies were incubated with 1% BSA-PBS instead of the first Ab. The sections were then incubated with HRP-conjugated goat anti-rabbit IgG (diluted to 1/500) for 1 h, at RT. After washing, the sections were developed with freshly prepared diaminobenzidine (DAB) chromogen solution (Sigma, Tokyo, Japan) for 7 min, counterstained with haematoxylin for 30 s, dehydrated in a series of ethanol dilutions, cleared in xylene, and mounted on glass coverslips. The images were viewed and photographed using a light microscope (Olympus, Tokyo, Japan). For computerized tomography (CT) analysis, a contrast agent was injected to the tail vein and CT images of the primary regions were obtained by Cosmo Scan (Rigaku, Tokyo, Japan).

### Tissue handling

HSC3-M3 cells (5 × 10^5^/35 μl PBS) were injected into the right edge of the tongue of male nude mice. Primary tumor mass and cervical lymph nodes were removed from NiCl_2_-fed and control mice 3 weeks later. The tissues were divided into two parts, one for paraffin-embedded sectioning and H&E staining, and the other for genomic DNA and total RNA extraction. Metastatic frequency was evaluated by detecting human β-globin gene according to the protocol described previously [42]. For real-time PCR and PCR array analysis, total RNA was extracted from the excised tongue tissues using the RNeasy mini kit (QIAGEN, Tokyo, Japan). For IP-Western blotting, the tumor mass were excised, minced and lysed with the cell lysis buffer. The IP-Western was performed as described above.

### Statistical analysis

Results are presented as mean ± SD from at least three independent experiments. Statistical differences were assessed using Student’s *t*-test, Welch’s test or Steel-Dwass test. A *P* value of *<* 0.05 was considered as significant.

## SUPPLEMENTARY MATERIALS FIGURE


