# Supplementary material for: Nickel chloride administration prevents the growth of oral squamous cell carcinoma
Source: Oncotarget. 2018 May 8;9(35):24109–21. doi: 10.18632/oncotarget.25313 (PMC5963632; doi:10.18632/oncotarget.25313)
Supplement: Supplementary file 1 [file oncotarget-09-24109-s001.pdf]

Nickel chloride administration prevents the growth of oral squamous cell carcinoma

SUPPLEMENTARY MATERIALS

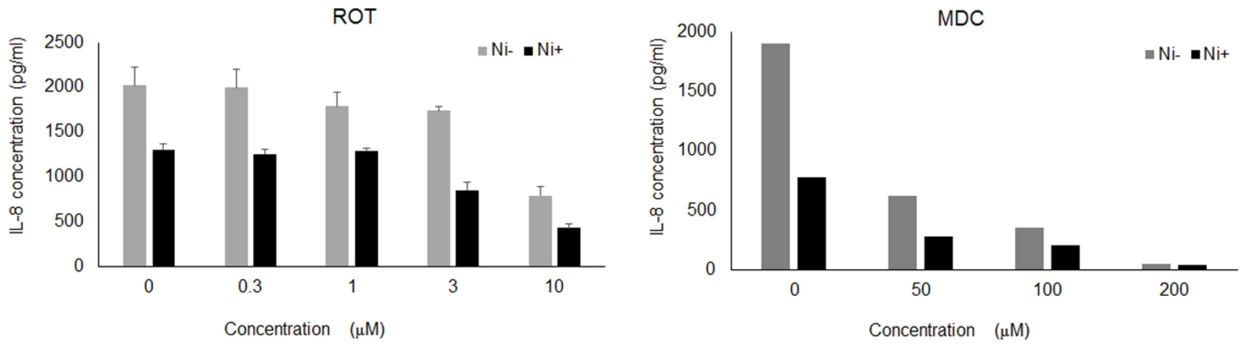

Supplementary Figure 1: Neither rottelerin (ROT) nor monodansylcadaverine (MDC) inhibited $NiCl_2$-induced IL-8 secretion. HSC3 cells were pre-treated with various concentrations of ROT (0, 0.3, 1, 3, 10 μM) or MDC (0, 50, 100, 200 μM) for 1 h. After treatment, cells were washed with PBS and further cultured for 18 h. The culture supernatants were harvested and subjected to IL-8 ELISA.
